# Advancing Measurable Residual Disease Detection in Pediatric BCP-ALL: Insights from Novel Immunophenotypic Markers

**DOI:** 10.3390/ijms26094282

**Published:** 2025-04-30

**Authors:** Alexandra Baldzhieva, Hasan Burnusuzov, Hristina Andreeva, Teodora Kalfova, Steliyan Petrov, Dobrina Dudova, Katya Vaseva, Marianna Murdjeva, Hristo Taskov

**Affiliations:** 1 Department of Medical Microbiology and Immunology “Prof. Dr. Elissay Yanev”, Faculty of Medicine, Medical University-Plovdiv, Vasil Aprilov Str. 15A, 4002 Plovdiv, Bulgaria; teodora.kalfova@mu-plovdiv.bg (T.K.); steliyan.petrov@mu-plovdiv.bg (S.P.); dobrina.dudova@mu-plovdiv.bg (D.D.); katya.vaseva@mu-plovdiv.bg (K.V.); mariana.murdzheva@mu-plovdiv.bg (M.M.); 2 Research Institute at Medical University-Plovdiv, Vasil Aprilov Str. 15A, 4002 Plovdiv, Bulgaria; hasan.burnusuzov@mu-plovdiv.bg (H.B.); hristina.andreeva@unn.no (H.A.); 3 Laboratory of Clinical Immunology, St. George University Hospital, Vasil Aprilov Str. 15A, 4002 Plovdiv, Bulgaria; hristo.taskov@mu-plovdiv.bg; 4 Department of Pediatrics and Medical Genetics, Faculty of Medicine, Medical University-Plovdiv, Vasil Aprilov Str. 15A, 4002 Plovdiv, Bulgaria; 5 Department of Immunology and Molecular Genetics, Laboratory Medicine Division, Diagnostic Clinic, University Hospital of North Norway, Hansine Hansens veg 67, 9019 Tromsø, Norway

**Keywords:** leukemia, flow cytometry, measurable residual disease, childhood B-cell precursor acute lymphoblastic leukemia, immunophenotyping, blasts

## Abstract

Measurable Residual Disease (MRD) assessment in pediatric acute lymphoblastic leukemia (ALL) is crucial for relapse prediction and treatment guidance. Multiparameter flow cytometry (MFC) enhances detection but faces limitations due to insufficient leukemia-associated immunophenotypes (LAIPs) and antigen modulation. This study explores new markers to improve MFC-based MRD detection in B-cell precursor ALL (BCP-ALL). Expression-patterns of seven aberrancy markers, i.e., CD44, CD304, CD73, CD86, CD123, CD99, CD58, and one B-cell maturation marker, CD22, were studied in 143 samples with leukemic-blasts from sixty-one childhood BCP-ALL patients and in hematogones of 20 non-leukemic bone marrow (BM) samples using fourteen-color MFC. The highest relative frequences of LAIPs amounted to 82.50%, reported for CD99 and CD58, followed by CD44 (81.10%), CD73 (76.20%), CD22 (73.40%), CD304 and CD86 (68.50%), while the lowest relative frequence was CD123 (44.40%). Differential expression of CD58, CD304, and CD73 in diagnostic samples was highly significant (*p* < 0.01) between pre-B-I, pre-B-II, immature B cells, and BCP-ALL blasts. In MRD-positive samples CD73 showed significantly high (*p* < 0.01) differential expression between all stages of hematogones and residual blasts, followed by CD304, CD58, and CD22. CD73 and CD304 were identified as the most reliable among the tested markers for distinguishing both diagnostic and MRD blasts from normal B cell precursors.

## 1. Introduction

Acute lymphoblastic leukemia (ALL) is the most common type of cancer in children. Despite significant advancements in treatment, relapse remains a major challenge in achieving long-term remission [1,2]. The ability to detect minimal/measurable residual disease (MRD) plays a critical role in identifying patients at high risk of relapse and guiding treatment decisions. MRD refers to a small population of leukemia cells that persist in a patient’s bone marrow (BM) or peripheral blood (PB) after chemotherapy or other treatments, often at levels too low for detection by conventional diagnostic methods [3,4].

The presence of MRD is considered one of the most important prognostic factors in ALL, with higher MRD levels strongly correlating with an increased risk of relapse. Consequently, accurate MRD detection is essential for tailoring treatment plans, determining the need for intensified therapy, or evaluating a patient’s eligibility for stem cell transplantation [4,5,6,7,8].

Traditional techniques for MRD detection, such as polymerase chain reaction (PCR) and cytogenetic methods, have certain limitations, particularly in sensitivity and their ability to provide continuous monitoring of disease progression. Multiparameter flow cytometry (MFC) has emerged as a powerful tool for MRD assessment due to its capacity to simultaneously analyze multiple cell surface markers and its high sensitivity for detecting residual leukemia cells at very low frequencies. By identifying leukemia-associated immunophenotypes (LAIPs) on the surface of leukemic cells, MFC achieves precise detection of residual disease with sensitivities as low as 0.01% or even 0.001% [9,10,11].

A study by Weng et al. demonstrated that an eight-color BCP-ALL MRD panel achieved a sensitivity of 1 in 10^4^ in 96% of cases, while reaching a sensitivity of 1 in 10^5^ in only 81.6% of cases, highlighting the need for further optimization of eight-color panels to improve sensitivity across all samples [12]. Similarly, a study by the EuroFlow Consortium, published in Blood, reported that their eight-color antibody panel for MRD detection in BCP-ALL achieved sensitivities comparable to those of real-time quantitative PCR (RQ-PCR), reaching as high as 10^−5^ in most cases. However, these results were consistent only when more than 4 million cells per sample were analyzed [13].

Advancements in flow cytometry (FC) have allowed for the inclusion of additional parameters, significantly enhancing sensitivity. This approach, involving the stepwise incorporation of more parameters, has culminated in the development of “Next-Generation Flow Cytometry” (NGF), which utilizes 10 or more parameters to achieve even greater sensitivity [9,14,15,16].

Despite its advantages, MFC-based MRD detection faces challenges in certain cases. Some patients with B-cell precursor acute lymphoblastic leukemia (BCP-ALL) do not exhibit sufficient or consistent LAIPs with commonly used markers, which can limit the effectiveness of MFC in identifying residual disease. Additionally, drug-induced antigen modulation—where chemotherapy or other treatments alter the expression of cell surface markers—can interfere with accurate MRD detection. These challenges underscore the need for a broader and more comprehensive set of markers to enhance the resolution and accuracy of MFC in MRD evaluation, ensuring reliable assessment even in cases with atypical or modulated immunophenotypic profiles [17].

A recent review by Verbeek et al. [18] emphasized the importance of incorporating novel markers to improve FC-MRD monitoring in BCP-ALL. Among these, CD58 has been shown to be overexpressed in over 99% of BCP-ALL cases, making it a promising marker for MRD detection. Similarly, a study by Coustan-Smith et al. [19] identified 22 differentially expressed markers, including CD44, CD73, and CD123, which are upregulated in BCP-ALL compared to normal B cells. Other markers, such as CD304, CD66c, and CD123, also demonstrated potential, with CD304 being overexpressed in 40–59% of BCP-ALL cases and CD123 showing strong prognostic value, particularly in cases with BCR-ABL1 and hyperdiploid karyotypes. These findings highlight an expanding list of candidate markers that could improve MRD sensitivity, disease monitoring, and risk stratification in BCP-ALL patients.

CD73, a glycosylphosphatidylinositol-anchored glycoprotein, has shown significant promise due to its role in immune modulation and its upregulation in B-ALL blasts [20,21]. Studies have demonstrated that CD73 expression increases in MRD-positive samples during treatment, making it a reliable marker for both diagnosis and monitoring treatment response. According to a recent study by Słota et al. [22], CD73 is a stable and effective marker for monitoring MRD, as 83.1% (147 of 177) of MRD-positive patients exhibited increased mean fluorescence intensity (MFI) by day 15 of treatment initiation. Increased CD73 expression was observed in 83.9% (26 of 31) of patients by day 33 of treatment.

A 2018 comparative study by Tembhare et al. further identified CD73 as a critical marker that should be routinely included in MRD monitoring panels due to its frequent expression in LAIPs [23]. Additionally, CD86 was highlighted as another robust marker to use alongside CD73 [23]. Supporting evidence indicates that CD73 is overexpressed in 90.4% of B-ALL cases, while CD86 is overexpressed in 60.9% [19].

The expression of CD123, the α-chain of the interleukin-3 receptor, has also gained attention due to its overexpression in leukemic stem cells, making it a potential diagnostic and therapeutic target [24,25]. However, CD123 expression did not correlate with MRD status at the end of induction or overall survival in some studies [25]. In contrast, a 2021 study by Zhiheng Li highlighted the importance of CD123 as an independent prognostic indicator associated with favorable outcomes in childhood B-ALL, where high CD123 expression correlated with improved survival rates [26].

CD304 is another promising marker, showing a significant difference in MFI between blasts and normal precursors. This marker enables clear differentiation of these populations and demonstrates notable stability in expression during therapy, relapse, and in cases with TEL-AML1 mutations. These features establish CD304 as an important prognostic marker and potential therapeutic target [27].

CD22 has also been recognized as a significant marker for distinguishing leukemic blasts from normal progenitors. It is expressed in the majority (60–90%) of B-ALL cases [28] and has become a target for immunotherapy [29]. Furthermore, its use in FC panels provides a distinct advantage for differentiating B-cell populations, particularly in cases where anti-CD19 therapies have been applied [16]. Additionally, CD22 is beneficial for identifying early BCPs that express CD22 but not yet CD19, offering significant value in detecting hematogones, especially in regenerating BM [30].

CD44, a marker involved in cell adhesion and migration, has also proven valuable in distinguishing leukemic cells from normal B-cell precursors [31]. A 2023 study by Abobakr et al. [32] found that CD44 was expressed in 91.6% of malignant cells in pediatric BCP-ALL and was associated with poor prognostic factors, including high peripheral blood blast counts, hepatomegaly, splenomegaly, and reduced physical activity.

CD99 is another marker that is upregulated in 50% of BCP-ALL cases compared to normal BCPs) [19]. Moreover, elevated CD99 expression is associated with high-risk subtypes, such as BCR-ABL1 and CRLF2-rearranged BCP-ALL. It also correlates with an increased likelihood of relapse, elevated MRD levels by day 29, and poorer overall survival outcomes [33].

Collectively, these markers, when incorporated into advanced FC panels, offer a more sensitive and accurate approach for MRD detection. They enhance the ability to identify residual leukemia cells, guide treatment decisions, and predict relapse risk in pediatric BCP-ALL patients.

In this study, we aimed to improve the sensitivity and accuracy of MRD detection in BCP-ALL using MFC with novel markers. To achieve this, we developed a 14-color FC panel by incorporating novel markers—CD22, CD86, CD73, CD99, CD44, CD304, and CD123—into the routinely used 8-color EuroFlow-based panel (CD45, CD19, CD20, CD38, CD10, CD34, CD58, and Syto41). The expression profiles of these new markers, along with CD58, were analyzed in 143 patient samples and 20 non-leukemic BM aspirates. This analysis identified the most effective markers for differentiating leukemic cells from normal BCPs and assessed their utility in MFC-based MRD analysis.

## 2. Results

### 2.1. Expression of the New Markers on Leukemic Blasts

Fluorescence intensity of markers was encoded in an ordinal scale according to the gradual increase in expression strength: 0—no expression (cases that express the indicated marker at levels lower than the lowest MFI measured in normal B-cell progenitors); 1—low (dim) expression; 2—moderate expression; 3—bright (over) expression (cases that express the indicated marker at levels higher than the highest MFI value recorded among normal B-cell progenitors). Expression grade 1, 2, and 3 was considered positive and zero expression was considered negative.

Among the patients tested, the highest relative proportion of positive expression (82.50%) was observed for CD99 and CD58. Next in descending order were CD44 (81.10%), CD73 (76.20%), CD22 (73.40%), CD304 and CD86 (68.50%), with CD123 having the lowest relative proportion of positive expression (44.40%).

**Figure 1:**
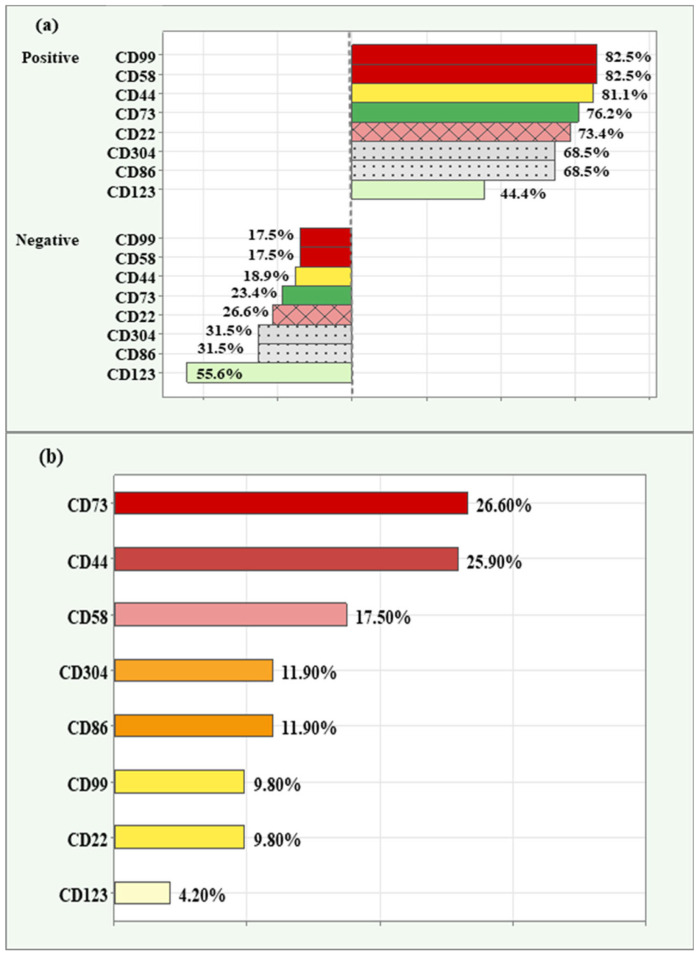
Relative proportions of positive and negative expression in novel markers (**a**); relative proportions of high/overexpression in novel markers (**b**) among the cases analyzed.

Similarly, the lowest relative proportion of negative expression was observed for CD99 and CD58 (17.50%), and the highest relative proportion of negative expression was reported for the marker CD123 (55.60%), (Figure 1a).

The relative proportion of high/overexpression (grade 3) is demonstrated in descending order in Figure 1b. Both CD73 (26.60%) and CD44 (25.90%) had the highest relative proportion of high expression. This was followed by CD58 (17.50%), CD304 and CD86 (11.90), CD99 and CD22 (9.80%). The lowest relative proportion was observed for CD123 (4.20%).

#### Distribution of Co-Expression Patterns Among the Three Most Frequently Expressed Markers

**Figure 2:**
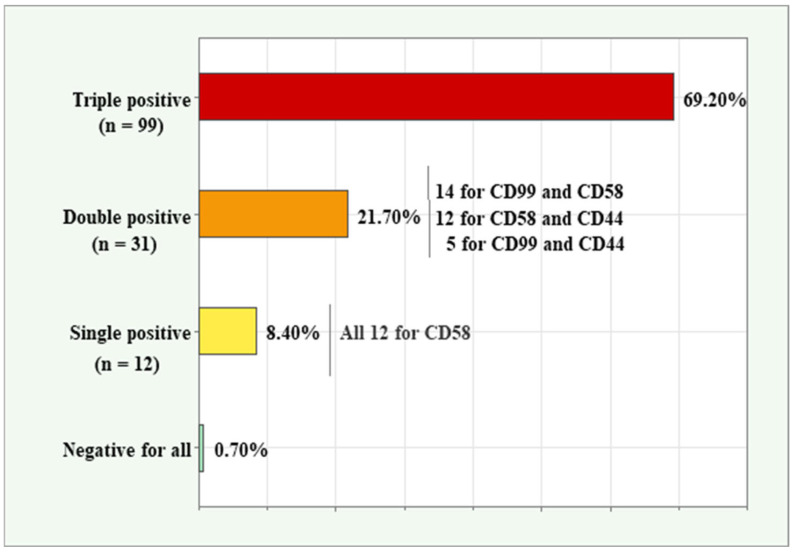
Co-expression patterns for the three markers (CD99, CD58, and CD44) with the highest relative expression in BCP-ALL samples.

The three aberrancy markers with the highest frequency of positive detection rates—CD99, CD58, and CD44—were analyzed together to determine their co-expression patterns in MRD-positive samples. The distribution of triple-positive, double-positive, single-positive, and negative samples is presented in Figure 2. Among all 143 samples, 99 (69.2%) were triple-positive, 31 (21.7%) were double-positive, 12 (8.4%) were single-positive, and one (0.7%) was negative for all three markers. Of the double-positive samples, 14 co-expressed CD99 and CD58, 12 co-expressed CD58 and CD44, and 5 co-expressed both CD99 and CD44. Notably, all 12 single-positive samples were associated with the CD58 marker, highlighting its distinct expression pattern in these cases.

### 2.2. MFI Comparison of the New Markers Across Diagnostic Blasts, Residual Blasts, and Normal B-Cell Precursors

For this analysis, normal B lymphocytes of interest in BM were categorized according to their stage of differentiation as follows:

1. Pre-B-I cells CD34+/CD10+high/CD19+low/CD20low to neg./CD45low (early hematogones, hematogones I),
2. Pre-B II cells CD45dim/CD34−/CD10+low/CD19+high/CD20low (late hematogones, hematogones II),
3. Immature B lymphocytes CD34−/CD10+/CD20++/CD19+, and
4. Mature B cells CD34−/CD10−/CD19+/CD20+.

Before conducting the statistical analysis, the normality of the distribution of novel markers within each group was assessed using Shapiro–Wilk test.

Due to lack of normal distribution (Shapiro–Wilk *p* < 0.05) in more than three of the five matched groups (Diagnostic/Residual blasts, Pre-B-1, Pre-B-2, immature B and mature B cells) for each of the markers, the mean trend was represented by the median and interquartile range (IQR). Kruskal–Wallis non-parametric test was used for statistical comparisons between groups, followed by pairwise comparisons using Bonferroni test.

Eight comparisons, one for each of the markers, were performed between diagnostic blasts and the four differential stages in normal B lymphocytes (Pre-B-1, Pre-B-2, immature B and mature B cells). Eight other comparisons were made between residual blasts and the stages of normal B cells. In total, 16 comparisons were performed using Kruskal–Wallis test and multiple pairwise comparisons using Bonferroni test (16 by 10 = 160). The results presented focus only on the comparisons relevant to the objectives of the current study.

#### 2.2.1. Comparison of MFI of the New Markers Between Diagnostic Blasts and Normal B Cells

**Figure 3:**
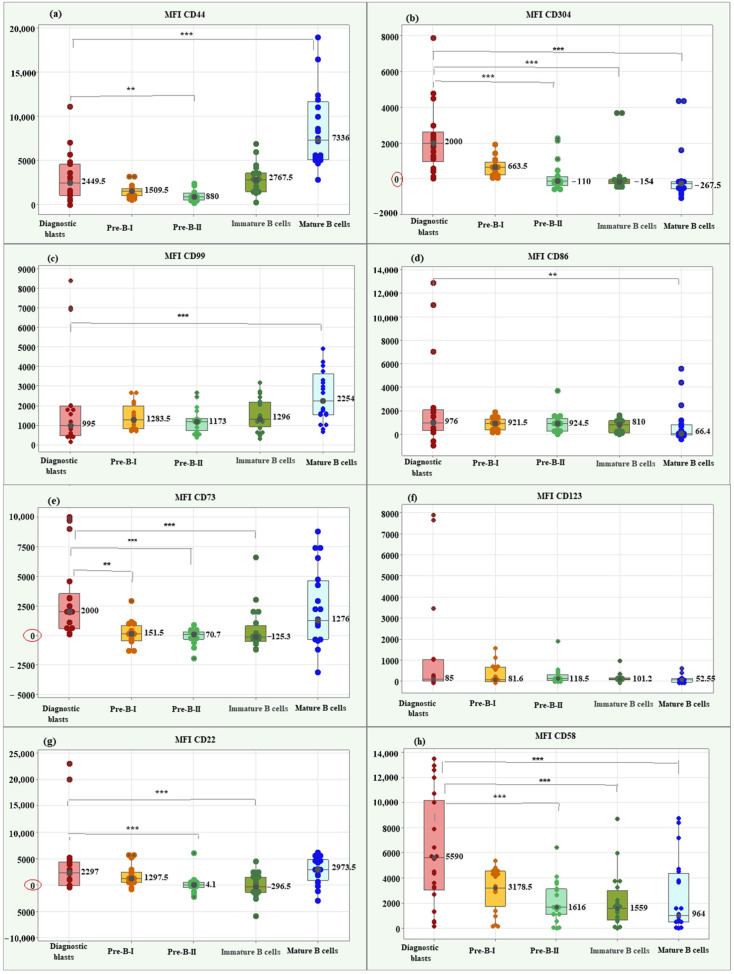
Box plots of individual values, medians, and interquartile ranges of MFI for the new markers in diagnostic BCP-ALL blasts. CD44 (**a**), CD304 (**b**), CD99 (**c**), CD86 (**d**), CD73 (**e**), CD123 (**f**), CD22 (**g**), and CD58 (**h**). MFI CD304 (**b**) and MFI CD58 (**h**) showed significantly higher value in diagnostic BCP-ALL blasts compared to Pre-B-II, immature B, and mature B cells. MFI CD73 (**e**) showed significantly higher value in diagnostic BCP-ALL blasts compared to Pre-B-I, Pre-B-II, and immature B cells. MFI CD22 demonstrated significantly higher value in blasts compared to late hematogones and immature B cells. ** Significant difference at *p* < 0.01; *** Significant difference at *p* < 0.001.

Kruskal–Wallis test showed a significantly higher median MFI CD44 value in diagnostic blasts (2449.50; IQR = 3533.75) compared to Pre-B-II cells (880; IQR = 788.25), *p* = 0.003. Diagnostic blasts showed a significantly lower median MFI for CD44 compared to mature B cells (7336; IQR = 6553.5), *p* < 0.001. Box plots for MFI CD44 illustrating the individual values, medians, and interquartile ranges in the matched groups are presented in Figure 3a.

Comparison between leukemic blasts at diagnosis and differentiation stages of normal B lymphocytes about MFI CD304 revealed a significant association between the marker values and the studied groups (*p* < 0.001). Three significant correlations of interest for the present study emerged using the Bonferroni test. The blasts showed a significantly higher median MFI CD304 value (2000; IQR = 1652.75) compared to Pre-B-II cells (−110; IQR = 236.9, *p* < 0.001), immature B cells (−154; IQR = 236.9, *p* < 0.001) and mature B cells (−267.5; IQR = 382.75 *p* < 0.001), (Figure 3b).

For CD99, the median MFI value in blasts (995; IQR = 1503.5) was notably lower than that observed in mature B cells, indicating a statistically significant difference (2254; IQR = 2095; *p* < 0.001), (Figure 3c).

The median MFI CD86 in blasts showed a significantly higher value (976; IQR = 1770.0) than the median of mature B cells (66.4; IQR = 864.15; *p* = 0.003), (Figure 3d).

There was a significant association between MFI CD73 values and matched groups (*p* < 0.001). The median MFI CD73 in blasts showed a significantly higher value (2000; IQR = 2957.5) than the medians of Pre-B-I (151.5; IQR = 1297.5; *p* = 0.002), Pre-B-II (70.7; IQR = 636.0; *p* = 0.001) and immature B cells (−125.3; IQR = 1324.75; *p* = 0.001), (Figure 3e).

Only MFI CD123 showed no significant association with the matched groups (*p* = 0.171). Figure 3f highlights the similar median values in blasts and normal B lymphocytes for this marker.

MFI CD22 showed a significant association with group type (*p* < 0.001). Of interest for the present study were two significant differences found by the Bonferroni test. Blasts showed a significantly higher median MFI CD22 value (2297; IQR = 1297.5) compared to Pre-B-II cells (−4.1; IQR = 976.75, *p* = 0.001) and concerning immature B cells (−296.5; IQR = 2886.7; *p* = 0.001), (Figure 3g).

Lastly, MFI CD58 showed a significant association with the study groups (Kruskal–Wallis *p* = 0.001). Blasts showed a significantly higher median MFI CD58 value (5590; IQR = 3178.5) compared to Pre-B-II cells (1616; IQR = 1559.0, *p* = 0.001), immature B cells (1559; IQR = 2337.0; *p* = 0.001), and mature B cells (964; IQR = 3851.5; *p* = 0.001), (Figure 3h).

#### 2.2.2. Comparison of MFI of the New Markers Between Residual Blasts and Normal B Cells

**Figure 4:**
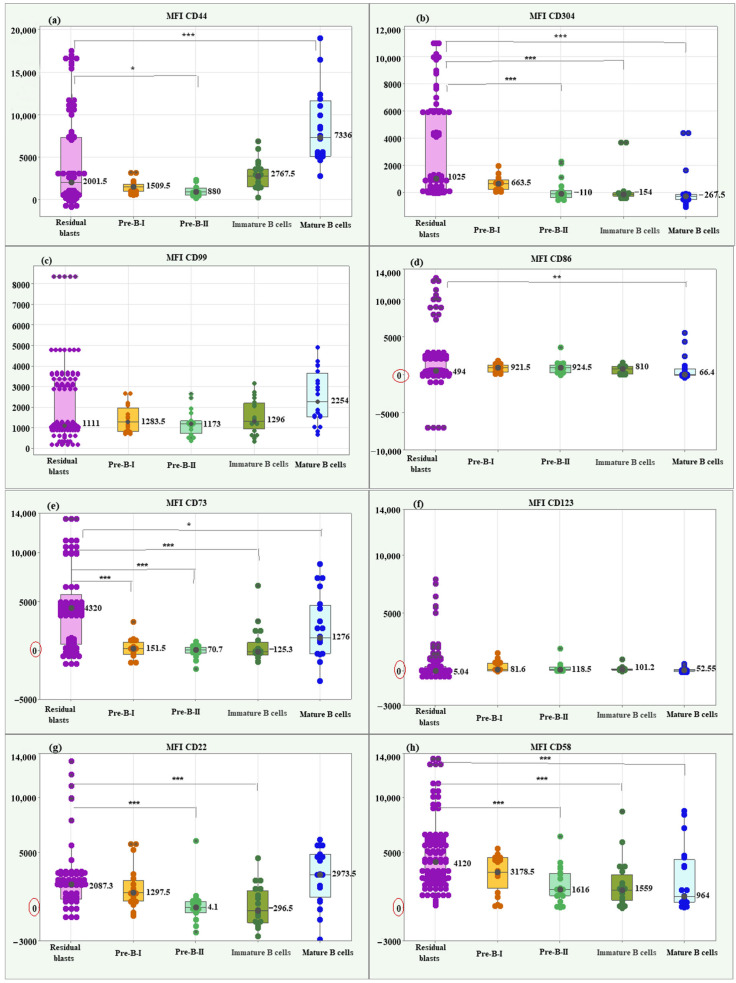
Box plots of individual values, medians, and interquartile ranges of MFI for the new markers in residual BCP-ALL blasts. CD44 (**a**), CD304 (**b**), CD99 (**c**), CD86 (**d**), CD73 (**e**), CD123 (**f**), CD22 (**g**), and CD58 (**h**). Residual BCP-ALL blasts displayed significantly higher MFI values for CD73 across all stages of normal B-cell differentiation, for CD58 and CD304—compared to Pre-B-II, immature B, and mature B cells, for CD22—to Pre-B-II and immature B cells, and for CD44—to Pre-B-II cells. * Significant difference at *p* < 0.05; ** Significant difference at *p* < 0.01; *** Significant difference at *p* < 0.001.

Residual blasts showed a significantly higher median MFI CD44 value (2001.5; IQR = 6849.5) compared to Pre-B-II cells (880; IQR = 788.25), *p* = 0.036, and a significantly lower median compared to mature B cells (7336; IQR = 6553.5), *p* < 0.001. Box plots of MFI CD44 illustrating individual values, medians, and interquartile ranges in the matched groups are presented in Figure 4a.

Regarding MFI CD304, the residual blasts showed a significantly higher median value (1025; IQR = 5785.0) compared to Pre-B-II cells (−110; IQR = 236.9, *p* < 0.001), immature B cells (−154; IQR = 236.9, *p* < 0.001) and mature B cells (−267.5; IQR = 382.75 *p* < 0.001), (Figure 4b).

The Kruskal–Wallis test showed no significant difference between residual blasts and normal B lymphocytes regarding MFI CD99 (*p* = 0.089), (Figure 4c).

The median MFI CD86 of residual blasts showed a significantly higher value (494; IQR = 2425.5) than the median of mature B cells (66.4; IQR = 864.15; *p* = 0.004, Figure 4d).

About the median MFI CD73, the residual blasts showed significantly higher levels (4320; IQR = 5060.0) compared to the differentiation stages of normal B cells: Pre-B-I (151. 5; IQR = 1297.5; *p* < 0.001), Pre-B-II (70.7; IQR = 636.0; *p* < 0.001), immature B cells (−125.3; IQR = 1324.75; *p* < 0.001), and mature B cells (1276; IQR = 4937.0; *p* = 0.034), (Figure 4e).

MFI CD123 showed no significant differences between residual blasts and normal BCPs (*p* = 0.215), (Figure 4f).

Residual blasts showed significantly higher median MFI CD22 (2087.3; IQR = 2262.0) compared to Pre-B-II cells (−4.1; IQR = 976.75, *p* < 0.001) and immature B cells (−296.5; IQR = 2886.7; *p* < 0.001), (Figure 4g).

Lastly, blasts had a significantly higher median MFI CD58 value (5590; IQR = 3178.5) compared to Pre-B-II cells (1616; IQR = 1559.0, *p* = 0.001), immature B cells (1559; IQR = 2337.0; *p* = 0.001) and mature B cells (964; IQR = 3851.5; *p* = 0.001), (Figure 4h).

In addition, a table was developed to present the number of cells contributing to the signal for each B-cell group, with a particular focus on the mean, median, and standard deviation (SD), (Table 1).

The Residual Blasts have a mean cell count of 2161.64 and a median of 2288, with a standard deviation of 267.52. The proximity of the mean and median values indicates a relatively normal distribution of cell counts within this group, suggesting stability in the number of residual blasts present. The low standard deviation reflects limited variability, implying that the population of residual blasts remains consistent across samples.

For the Pre-B-I cells, the mean is 8537.10, with a median of 13,128.00 and a large standard deviation of 22,401.90. The disparity between the mean and median suggests a skewed distribution, likely with a subset of samples exhibiting substantially higher cell counts, contributing to the elevated mean. The high standard deviation indicates considerable variability in the number of Pre-B-I cells across different samples.

Pre-B-II cells display an exceptionally high mean cell count of 213,539.75 but a lower median of 28,774.00, alongside a standard deviation of 37,162.67. This significant difference between the mean and median indicates a highly skewed distribution, where a few samples likely contain very high counts of Pre-B-II cells, inflating the mean.

The Immature B cells show a mean count of 32,416.30, with a median of 18,517.50 and a standard deviation of 33,343.77. The mean is influenced by a few samples with high cell counts, as indicated by the larger standard deviation compared to the median, ands all this leads to a wide range of counts across samples.

Lastly, the Mature B cells exhibit a mean cell count of 56,374.95, with a median of 43,010.00, and a standard deviation of 58,503.87. The substantial standard deviation indicates a high degree of variability in the number of mature B cells across samples. The difference between the mean and median suggests that some samples may possess particularly high counts, indicative of active maturation processes or responses to treatment.

**Figure 5:**
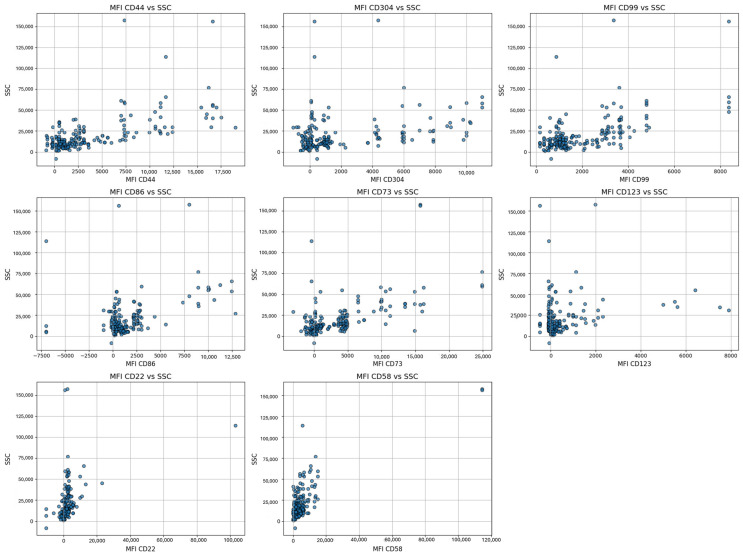
Visualization of the cell clusters of residual blasts based on mean fluorescence intensity (MFI) for specific surface markers (CD44, CD304, CD86, CD73, CD123, CD22, CD58, and CD99) against side scatter (SSC), reflecting variations in cell complexity and activation states.

**Figure 6:**
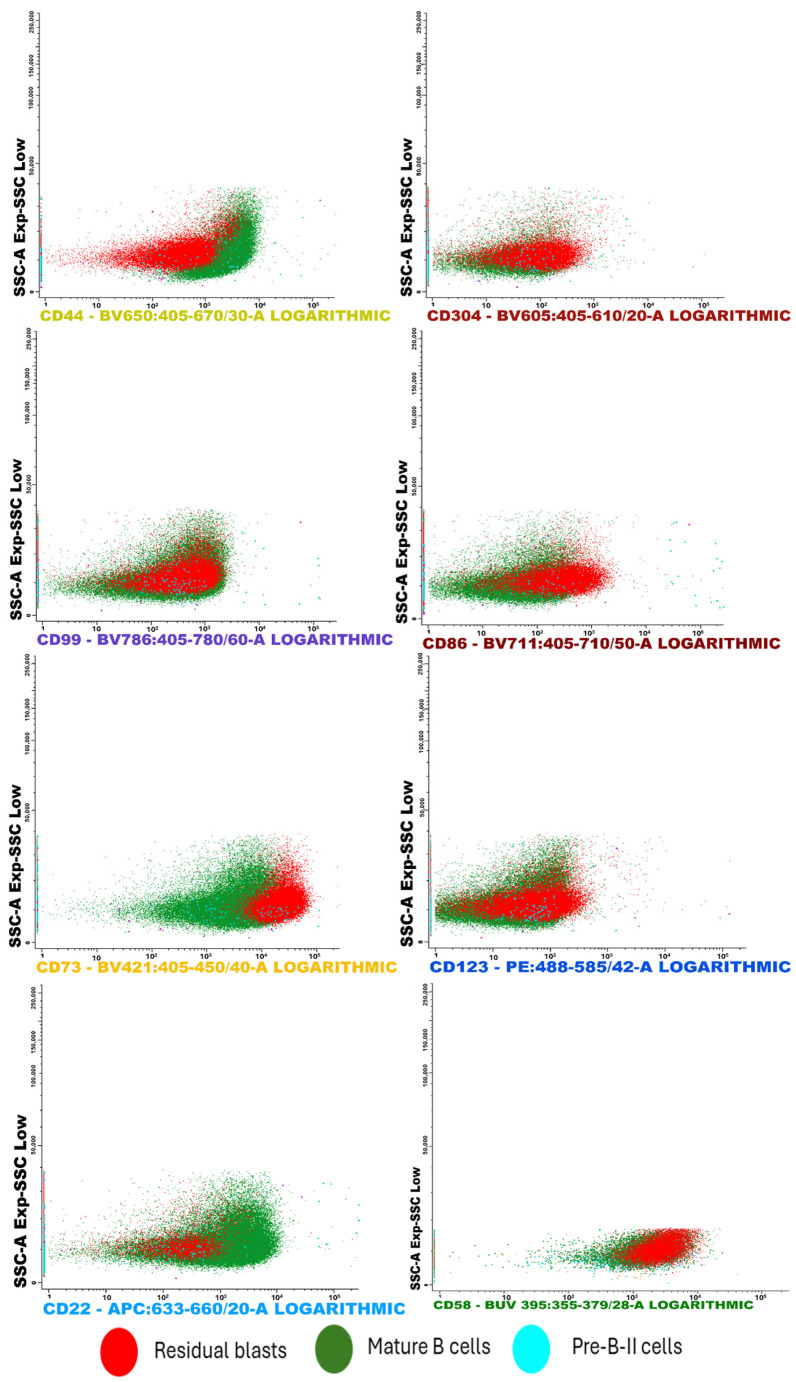
This FC analysis displays scatter plots illustrating the separation of cell clusters from a bone marrow aspirate. Notably, the sample showed a low presence of Pre-B I and immature B lymphocytes. Among the most effective CD markers for detecting residual blasts, CD73 exhibited strong positivity in leukemic cells compared to both mature B cells and Pre-B II cells. Similar trends were observed with the markers CD304 and CD58.

Moreover, the scatter plots offer a detailed visualization of cell clusters based on mean fluorescence intensity (MFI). They present specific surface markers—including CD44, CD304, CD86, CD73, CD123, CD22, CD58, and CD99—plotted against side scatter (SSC), (Figure 5). Additionally, flow cytometry analysis illustrates the separation of cell clusters from a bone marrow aspirate of a patient with positive MRD (Figure 6).

### 2.3. Multiple Logistic Regression Analysis of the Novel Biomarkers for MRD Risk Prediction

An initial multiple logistic regression analysis was conducted using full model to evaluate the influence of eight predictors (CD44, CD304, CD99, CD86, CD73, CD123, CD22, CD58) on the risk of MRD (MRD: 0 = no risk, 1 = at risk). The null model, which included only the constant, yielded a baseline probability of 39.8% for MRD = 1, with an overall accuracy of 60.2% (sensitivity 0.0%; specificity 100.0%, Table 2). The full model, incorporating all predictors, significantly improved the fit, reducing the −2 Log Likelihood from 150.000 (null model) to 101.944, while the Nagelkerke R^2^ reached 0.767, explaining 76.7% of the variance in the dependent variable (Table 2).

The coefficients of the full model revealed that five of the eight predictors were statistically significant (*p* < 0.05) (Table 3). CD58 exhibited the strongest effect on risk (OR = 21.911, 95% CI: 5.033–95.389, *p* < 0.001), followed by CD304 (OR = 3.245, 95% CI: 2.038–5.167, *p* < 0.001) and CD73 (OR = 2.997, 95% CI: 2.035–4.415, *p* < 0.001), all of which positively influenced the likelihood of MRD. CD44 (OR = 0.397, 95% CI: 0.196–0.804, *p* = 0.010) and CD123 (OR = 0.575, 95% CI: 0.364–0.910, *p* = 0.018) had a negative coefficient, indicating that a decrease in their expression was associated with the classification of cases as MRD. CD99, CD86, and CD22 were not significant (*p* > 0.05). The classification accuracy of the full model reached 90.0%, with a sensitivity of 92.6% and a specificity of 86.3%, indicating a high ability to identify at-risk cases (Table 2).

Following the full model, a stepwise multiple logistic regression analysis using the Forward Selection method was conducted to optimize the model by sequentially adding significant predictors. The analysis progressed through five steps, with predictors added as follows: CD304 (Step 1), CD73 (Step 2), CD58 (Step 3), CD44 (Step 4), and CD123 (Step 5). Model fit improved progressively, as evidenced by the reduction in −2 Log Likelihood from 194.315 in Step 1 to 105.346 in Step 5, and the Nagelkerke R^2^ increased from 0.425 to 0.757, explaining 75.7% of the variance in the dependent variable by Step 5 (Table 4). The Hosmer–Lemeshow goodness-of-fit test indicated poor fit in Step 1 (χ^2^ = 41.847, df = 6, *p* < 0.001), but acceptable fit in Steps 2–5 (*p* > 0.05), with Step 4 demonstrating the best fit (χ^2^ = 2.873, df = 8, *p* = 0.942) (Table 4).

Classification accuracy also improved across steps, rising from 77.6% in Step 1 to a peak of 89.1% in Step 4, before slightly decreasing to 88.6% in Step 5 (Table 4). In Step 1, the model, which included only CD304 (OR = 2.749, *p* < 0.001), achieved a high sensitivity of 87.6% but a lower specificity of 62.5%, indicating a tendency to overpredict risk. The addition of CD73 in Step 2 (OR = 2.190, *p* < 0.001) increased specificity to 76.3%, improving overall accuracy to 82.1%. Further inclusion of CD58 in Step 3 (OR = 16.401, *p* < 0.001) and CD44 in Step 4 (OR = 0.458, *p* = 0.007) continued to enhance the model, with Step 4 achieving the highest accuracy (89.1%), sensitivity (91.7%), and specificity (85.0%) (Table 4). The coefficients for Step 4 confirmed that CD58 had the strongest effect on risk (OR = 18.303, *p* < 0.001), followed by CD304 (OR = 2.911, *p* < 0.001) and CD73 (OR = 2.582, *p* < 0.001), all of which significantly increased the likelihood of MRD = 1 (Table 5). The addition of CD123 in Step 5 (OR = 0.575, *p* = 0.012) did not improve the model, as the overall accuracy slightly decreased to 88.6%, with a reduction in specificity to 83.8%, suggesting that CD123 may introduce noise or redundancy.

From the results presented above, the following conclusions can be drawn. The multiple logistic regression analyses provided valuable insights into the predictors of the MRD. The full model, which included all eight predictors, achieved a high classification accuracy of 90.0%, with a sensitivity of 92.6% and a specificity of 86.3%, explaining 76.7% of the variance (Nagelkerke R^2^ = 0.767). Significant risk factors identified included CD58 (OR = 21.911), CD304 (OR = 3.245), and CD73 (OR = 2.997), while CD44 and CD123 exhibited protective effects. However, the inclusion of non-significant predictors (CD99, CD86, CD22) suggested potential overfitting.

The Forward Selection method optimized the model by focusing on the most relevant predictors, achieving a peak accuracy of 89.1% in Step 4 with a sensitivity of 91.7% and a specificity of 85.0%. This model retained only CD44, CD304, CD73, and CD58, confirming their importance as key predictors. The Nagelkerke R^2^ in Step 4 was 0.736, explaining 73.6% of the variance, and the Hosmer–Lemeshow test indicated excellent fit (*p* = 0.942). The slight decrease in accuracy to 88.6% in Step 5 upon adding CD123 (OR = 0.575, *p* = 0.012) suggests that this predictor does not substantially improve the model and may be excluded in practical applications. Compared to the Full model, the Forward Selection approach (Step 4) provided a more parsimonious model with comparable predictive performance (89.1% vs. 90.0% accuracy), making it more suitable for clinical use where simplicity and interpretability are prioritized. Both models demonstrated high sensitivity, which is critical for identifying at-risk cases in a medical context, but the Forward Selection model (Step 4) offers a better balance of accuracy, fit, and efficiency.

Future studies should validate these findings in larger cohorts, explore the biological mechanisms underlying the strong effect of CD58, and consider additional diagnostic metrics, such as the area under the ROC curve, to further assess the models’ discriminatory power.

### 2.4. Variable Importance Analysis for MRD Status Prediction Using Random Forest

To identify the most influential biomarkers for predicting minimal residual disease (MRD) status, a Random Forest classification model was applied to a dataset comprising 201 patients, with MRD as the binary target variable (1 = diseased, 0 = healthy) and eight biomarkers as predictors: CD304, CD73, CD58, CD44, CD86, CD123, CD99, and CD22. The model was trained using 100 trees, with the number of features considered at each split set to the default value of log_2_(8) + 1 = 4 and evaluated using 10-fold cross-validation to ensure robust performance estimates.

The RF model achieved high classification performance, as detailed in Table 5. The overall accuracy was 93.33%, with a 95% confidence interval (CI) of (0.838, 0.9815). Sensitivity and specificity were 87.50% and 97.22%, respectively, indicating a strong ability to distinguish between MRD-positive and MRD-negative cases. The Kappa statistic (0.8592) confirmed excellent agreement beyond chance. McNemar’s test (*p* = 0.6171) indicated no significant bias in misclassification errors.

**Figure 7:**
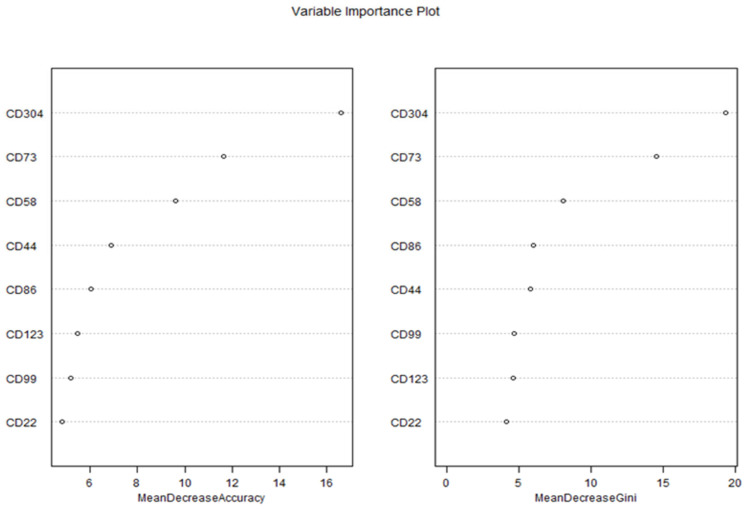
Variable importance plot of biomarkers for predicting MRD status using Random Forest.

Feature importance was assessed using two metrics: Mean Decrease in Accuracy (MDA) and Mean Decrease in Gini (MDG), which were visualized in a Variable Importance Plot (Figure 7).

The Variable Importance Plot revealed consistent rankings across both metrics, highlighting the relative contributions of each biomarker to the model’s predictive performance. According to the MDA metric, CD304 emerged as the most important predictor, with an MDA value of 16.6, indicating that permuting CD304 values resulted in the largest reduction in model accuracy. This was followed by CD73 (MDA = 12.5), CD58 (MDA = 11.5), and CD44 (MDA = 10.5), which also demonstrated substantial contributions to the model’s accuracy. In contrast, CD86 (MDA = 9.5), CD123 (MDA = 8.5), CD99 (MDA = 7.5), and CD22 (MDA = 6.5) exhibited lower importance, suggesting a more limited role in distinguishing between diseased and healthy patients.

The MDG metric, which quantifies the contribution of each biomarker to the reduction in node impurity (Gini index) across all trees, corroborated these findings. CD304 again ranked highest with an MDG value of 19.4, followed by CD73 (MDG = 14.6), CD58 (MDG = 8.1), and CD44 (MDG = 5.8). The remaining biomarkers—CD86 (MDG = 6.0), CD99 (MDG = 4.7), CD123 (MDG = 4.6), and CD22 (MDG = 4.1)—showed progressively smaller contributions to node purity, consistent with their lower MDA rankings. The strong agreement between MDA and MDG rankings underscores the robustness of these results, as both metrics independently identified CD304, CD73, and CD58 as the top three predictors.

These results show that the Random Forest model demonstrated excellent predictive performance in classifying MRD status using the selected biomarkers. The high accuracy, specificity, and sensitivity suggest that the model can be effectively applied for MRD detection. Among the biomarkers, CD304 exhibited the highest predictive value, indicating its potential as a key marker for MRD assessment. CD73 and CD58 also contributed significantly to model performance, while CD123 and CD22 showed lower importance.

These findings align with previous studies highlighting the role of CD304 in hematological malignancies. The strong predictive performance suggests that this marker, alongside CD73 and CD58, could serve as valuable targets for refining MRD detection protocols. Further validation in larger patient cohorts is necessary to confirm these results and evaluate their clinical applicability.

### 2.5. Determination of Optimal Threshold Values of the New Markers with the Highest Level of Differentiation of Malignant from Normal B Cells

For all new markers, screening with ROC curve was performed to identify those with the best diagnostic reliability in distinguishing malignant cells (diagnostic and residual) from the four differential stages of normal B cells. Table 6 and Table 7 summarize the results of all 64 ROC curve analyses.

In general, an AUC of 0.5 suggests no discrimination (i.e., ability to diagnose patients with and without the disease or condition based on the test), 0.7 to 0.8 is considered acceptable, 0.8 to 0.9 is considered excellent, and more than 0.9 is considered outstanding [34].

As shown in Table 6, MFI CD73 demonstrated the highest reliability in distinguishing diagnostic blasts from early hematogones (AUC = 0.855) and late hematogones (AUC = 0.920). Additionally, MFI CD73 proved to have excellent diagnostic reliability for differentiating immature B lymphocytes (AUC = 0.850). On the other hand, MFI CD304 showed the highest accuracy in distinguishing diagnostic blasts from immature B cells (AUC = 0.905) and mature B cells (AUC = 0.895) but also achieved excellent and outstanding reliability for Pre-B-I (AUC = 0.802) and Pre-B-II (AUC = 0.910) cells, respectively. MFI CD58 was also presented as an excellent discriminative indicator between blasts and late hematogones (AUC = 0.801).

Concerning residual blasts, the same two markers showed the best diagnostic reliability for identification from healthy B lymphocytes. MFI CD73 demonstrated the highest reliability in distinguishing residual blasts from Pre-B-I (AUC = 0.836) and Pre-B-II (AUC = 0.864). MFI CD304 showed the highest accuracy in differentiating residual blasts from immature B cells (AUC = 0.914) and mature B cells (AUC = 0.900). The latter also illustrated excellent discriminative value regarding hematogones II (0.872), as well as MFI CD22 (AUC = 0.849, Table 7).

The presented analyses identified MFI CD73 and MFI CD304 as the most reliable of the additional markers for determining threshold values distinguishing blasts from normal B cells. MFI CD73 emerged as the leading marker in distinguishing blasts (both diagnostic and residual) from Pre-B-I and Pre-B-II cells. MFI CD304 showed the best diagnostic reliability in differentiating blasts (both diagnostic and residual) from immature and mature B cells as well as from hematogones I and II, with slightly better diagnostic reliability in differentiating late versus early hematogones. Regarding the late hematogones, MFI CD58 demonstrated excellent ability to discriminate them from diagnostic blasts, while MFI CD22 proved to be an excellent marker for identifying them from residual blasts.

#### 2.5.1. Threshold Values of MFI CD73 in Distinguishing Diagnostic and Residual BCP-ALL Blasts from Pre-B-I and Pre-B-II Cells

MFI CD73 showed a reliability of 85.50% (AUC = 0.855) in distinguishing diagnostic blasts from Pre-B-I, with a threshold value > 441, which was characterized by 81.82% sensitivity and 75.00% specificity, a positive predictive value of 78.30% and a negative predictive value of 78.90%. The results regarding MFI CD73 in distinguishing diagnostic blasts from Pre-B-II cells showed a high diagnostic reliability of 92.00% (AUC = 0.920), with a threshold value > 444, sensitivity of 81.82%, specificity of 95.00%, positive predictive value of 94.70%, and negative predictive value of 82.60%.

Statistics for the diagnostic role of MFI CD73 in differentiating residual blasts from Pre-B-I cells showed a reliability of 83.60% (AUC = 0.836), with a threshold value > 2930, which was characterized by 66.94% sensitivity and 100% specificity, a positive predictive value of 100% and a negative predictive value of 33.30%. MFI CD73 to differentiate residual blasts from Pre-B-II cells showed a diagnostic reliability of 86.40% (AUC = 0.864), with a threshold value > 886, sensitivity of 73.55%, specificity of 100%, positive predictive value of 100% and negative predictive value of 38.50%.

**Figure 8:**
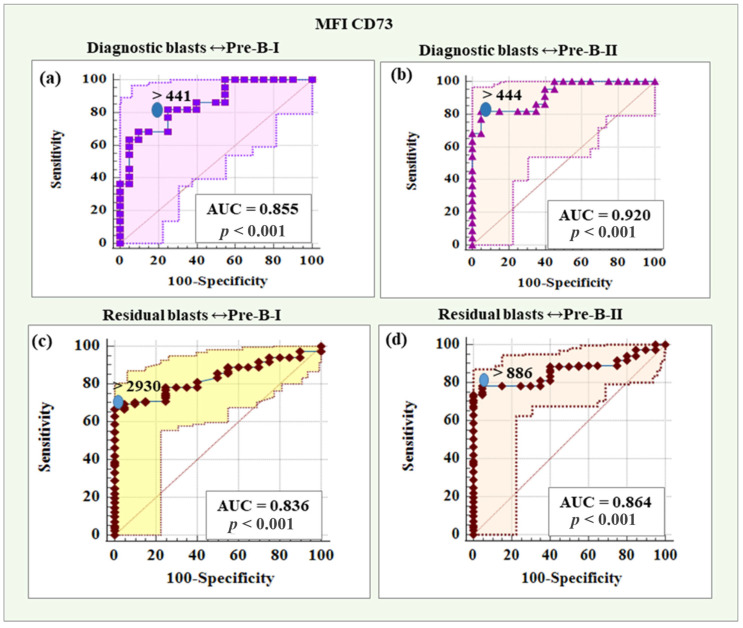
ROC curves for the diagnostic potential of MFI CD73 in differentiating BCP-ALL blasts from Pre-B-I and Pre-B-II cells. (**a**) ROC curve for CD73 in differentiating diagnostic BCP-ALL blasts from Pre-B-I lymphocytes. (**b**) ROC curve for CD73 expression levels in discriminating diagnostic BCP-ALL blasts from Pre-B-II cells. (**c**) ROC curve demonstrating the diagnostic potential of CD73 in differentiating residual blasts from Pre-B-I lymphocytes. (**d**) ROC curve for CD73 expression levels in discriminating minimal residual disease (MRD) blasts from Pre-B-II cells.

Figure 8 shows the ROC curves for the diagnostic potential of MFI CD73 in differentiating blasts from Pre-B-I and Pre-B-II cells.

#### 2.5.2. Threshold Values of MFI CD304 in Distinguishing Diagnostic and Residual BCP-ALL Blasts from Immature and Mature B Cells

MFI CD304 demonstrated a reliability of 90.5% (AUC = 0.905), with a threshold value > 114, a sensitivity of 90.91%, a specificity of 90.00%, a positive predictive value of 90.90%, and a negative predictive value of 90.00% in distinguishing diagnostic blasts from immature B cells.

Based on MFI CD304, the reliability of differentiating diagnostic blasts from mature B cells was 89.50% (AUC = 0.895), with a threshold value > −112, sensitivity was 100%, specificity was 85.00%, positive predictive value was 88.00%, and negative predictive value was 100%.

The diagnostic reliability of MFI CD304 in distinguishing residual blasts from immature B was 91.40% (AUC = 0.914), with a threshold value > −14.6, which was characterized by a 97.52% sensitivity and an 80% specificity, a positive predictive value of 96.70%, and a negative predictive value of 84.20%. Regarding the differentiation of residual blasts from mature B cells, MFI CD304 showed a diagnostic reliability of 90.00% (AUC = 0.900), with a threshold value > −112, a sensitivity of 100%, a specificity of 85.00%, a positive predictive value of 97.60% and a negative predictive value of 100%.

**Figure 9:**
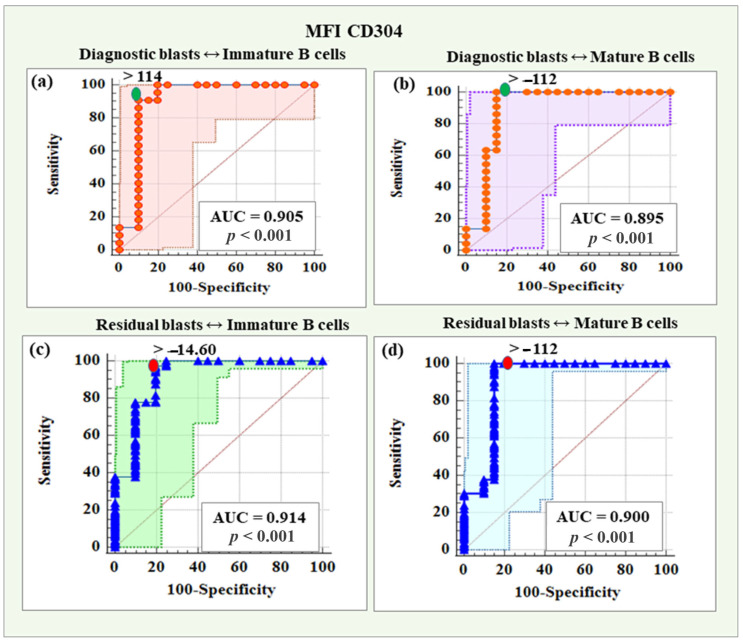
ROC curves for the diagnostic potential of MFI CD304 in distinguishing diagnostic and residual blasts from immature and mature B cells. (**a**) ROC curve for CD304 in differentiating diagnostic BCP-ALL blasts from immature B lymphocytes. (**b**) ROC curve for CD304 expression levels in discriminating diagnostic BCP-ALL blasts from mature B cells. (**c**) ROC curve demonstrating the diagnostic potential of CD304 in differentiating residual blasts from immature B lymphocytes. (**d**) ROC curve for CD304 expression levels in discriminating minimal residual disease (MRD) blasts from mature B cells.

ROC curves for the diagnostic potential of MFI CD304 in distinguishing BCP-ALL blasts from immature and mature B cells are presented in Figure 9.

## 3. Discussion

Comparing our findings with previously reported data on novel markers in BCP-ALL reveals several alignments and discrepancies. CD99 and CD58 exhibited the highest relative proportions of positive expression, with 82.5% of cases showing positivity. The frequency of CD58 positivity in our cohort aligns with Verbeek’s review [18], which reported over 90% positivity, and other studies citing positivity rates of up to 93.5% [35]. CD58 demonstrated excellent diagnostic reliability in distinguishing late hematogones from BCP-ALL blasts in our cohort. Burnusuzov et al. further confirmed the stability of CD58 expression despite immunophenotypic modulation [36]. Moreover, CD58 overexpression has been associated with better prognosis in pediatric B-ALL cases [37,38]. Notably, single-positive samples in our study that co-expressed the three highest-expressed markers were exclusively positive for CD58, underscoring its stability and suggesting it could serve as a standalone marker when other markers are underexpressed or absent, enhancing diagnostic reliability.

While CD99 was frequently detected in our cohort (82.5%), its expression levels were predominantly low, with only 10% of positive cases showing bright expression. Coustan-Smith reported a prevalence of approximately 50% for CD99 in B-ALL cases [19]. Elevated CD99 expression, especially in high-risk BCP-ALL subtypes with BCR-ABL1 and CRLF2 mutations, correlates with poor prognostic factors such as relapse, higher MRD positivity by day 29, and reduced overall survival [33]. These findings highlight the potential of CD99 as a prognostic biomarker, aiding in patient stratification and therapeutic decision-making.

For CD44, our study showed a lower frequency of underexpression (19%) compared to Coustan-Smith’s study (28%) [19], while Tembhare reported an even lower underexpression rate of 1.1% [23]. Abobakr et al. (2023) observed positive CD44 expression in 91.6% of malignant cells in pediatric BCP-ALL and associated it with poor prognostic factors such as high peripheral blood blast counts, hepatomegaly, and splenomegaly [32]. CD44 was linked to aggressive disease features, increased relapse risk, and unfavorable clinical outcomes, emphasizing its value as a prognostic marker for identifying high-risk patients and optimizing treatment strategies.

Our analysis revealed that the majority of samples (69.2%) exhibited triple positivity for CD58, CD99, and CD44, which had the highest relative expression. Double-positive samples accounted for 21.7%, with CD99-CD58 being the most frequent combination. The prevalence of triple- and double-marker positivity underscores the combined diagnostic value of these markers. The low proportion of marker-negative samples (0.7%) highlights the comprehensive detection capability of this marker combination, further supporting their potential in MRD detection panels to minimize the risk of missing leukemic cells due to variable marker expression.

CD73 was the next most frequently expressed marker in our cohort (76.2%), with substantial overexpression in 26.6% of cases. Our results are consistent with previous studies [18,19,39,40,41]. Compared to Tembhare’s findings [23], CD73 positivity was similar (76.2% vs. 77%), but higher than Coustan-Smith’s report (54.5%), [19]. Sedek et al. highlighted the strong diagnostic potential of CD73, noting its stable expression across MRD-Day 15 and early follow-up MRD time points (95%) [39]. Moreover, CD73 exhibited aberrant expression (16%) at MRD-Day 15 despite being negative at diagnosis, further underscoring its diagnostic utility [39].

CD304 and CD86 exhibited moderate positivity rates (68.5%) in our cohort, with lower frequencies of high expression (11.9%). Previous studies have reported CD304 positivity rates between 40 and 59% [18,27,39,42]. CD304 is involved in processes like angiogenesis and immune responses and has been identified as a potential target for anti-leukemia therapies and an MRD marker in B-ALL [27,39,43]. Studies, including those by the EuroFlow Consortium, confirmed CD304 overexpression as a significant MRD marker, suggesting its dual role as a prognostic factor and therapeutic target [13]. Research has shown that while CD304 expression may decrease in some BCP-ALL cases, it remains aberrantly positive in 63%, affirming its value in MRD detection. Combined with CD73, CD304 can distinguish normal BCP cells from leukemic blasts in about one-third of patients, outperforming markers like CD123 [13,39].

Mansour et al. (2023) demonstrated significantly higher CD86 MFI levels in pediatric ALL patients compared to healthy controls [44]. In our cohort, mature B-lymphocytes from non-leukemic controls had median MFI levels of 66.4, whereas leukemic blasts had significantly higher MFI values (976, *p* < 0.01), supporting the diagnostic relevance of CD86. Elevated levels of this marker were associated with poor prognosis, including higher relapse rates and mortality, potentially due to its role in inhibiting apoptosis and enhancing leukemic cell survival [44,45,46]. CD86 also showed stability between MRD-Day 15 and early follow-up MRD time points (71%), with 18% of cases acquiring positivity at MRD-Day 15 after being negative at diagnosis, emphasizing its diagnostic value [39].

CD123 had the lowest positive expression rate in our cohort (44.4%), aligning with Coustan-Smith’s (50.7%) and Theunissen’s findings (55%) [13,19]. As CD123 is typically absent or expressed at low levels on normal BCP cells, it is a valuable biomarker for distinguishing malignant BCP-ALL cells. While CD123 had the lowest positivity and overexpression rates (4.2%) in our study, its specific expression in certain cases offers targeted diagnostic and therapeutic potential.

CD22 was expressed in 73.4% of patients, which is slightly lower than the >90% prevalence reported in other studies [18]. Its high expression underscores its importance in diagnostic panels, especially for patients undergoing CD19-targeted therapies where CD19 may be lost. Including CD22 in diagnostic panels enhances the detection of early BCP cells that express CD22 but not yet CD19, which is crucial for identifying all BCP cells in regenerating BM [13,30]. CD22 is now commonly included in next-generation FC panels alongside CD19 for improved diagnostic accuracy [47].

Sedek et al. [39] conducted one of the most comprehensive studies on MFI levels of CD73, CD86, and CD304 in BCP-ALL, confirming our findings. Their data showed low expression of CD86 and CD304 in normal pre-B-II and immature B cells, with CD73 expressed slightly less frequently (66%) compared to our cohort (76.2%). Despite occasional decreases, CD304 remained a robust MRD marker, and CD73 showed the highest stability and MFI elevation, supporting its role in tracking leukemic blasts. In our study the Random Forest analysis identified CD304 as the most critical biomarker for predicting MRD status, followed by CD73 and CD58, as evidenced by their consistently high rankings in both Mean Decrease in Accuracy and Mean Decrease in Gini metrics. These findings highlight the potential of CD304, CD73, and CD58 as key predictors in the diagnostic assessment of MRD, offering insights into the underlying immunological mechanisms of the disease. Combining CD73 and CD304 antibodies, along with acquiring ≥4 million cells per tube, and applying threshold MFI values derived from our results—MFI CD73 > 2930 and >886 for distinguishing diagnostic and residual BCP-ALL blasts from Pre-B-I and Pre-B-II cells, respectively, and MFI CD304 > 14.6 and >112 for differentiating diagnostic and residual BCP-ALL blasts from immature and mature B cells, respectively, could achieve MRD-positive detection rates comparable to PCR methods, significantly improving differentiation between normal BCP cells and leukemic blasts in one-third of patients, outperforming markers like CD123 [39]. Biomarkers such as CD22, CD123, and CD99, which exhibited lower importance, may have limited predictive value in this context but could still hold biological significance in other disease subsets or in combination with additional variables.

The evolution of personalized medicine in the treatment of BCP-ALL hinges on the integration of biomarkers for early detection of MRD. By employing specific markers such as CD58, CD73, and CD304, more precise risk stratification can be achieved, enabling tailored treatment decisions based on individual patient profiles.

Early MRD detection is critical, as it allows timely intervention, adjustment of therapy intensity, and monitoring of treatment response. For instance, the stability and positive prognostic association of CD58 make it a valuable tool for identifying high-risk patients, while CD73’s strong diagnostic utility enhances the reliability of MRD assessments. Furthermore, biomarkers like CD304 not only assist in detection but also present opportunities for therapeutic targeting, representing a dual benefit in patient care.

The systematic implementation of a multi-marker approach within clinical protocols will be essential in the shift toward personalized medicine. By standardizing assessments and training clinicians, treatment pathways can be optimized with improved patient outcomes in BCP-ALL. Continued research will further elucidate the roles of these biomarkers, solidifying their place in future therapeutic strategies and enhancing the overall landscape of personalized healthcare in oncohematology [48,49,50].

In summary, our study validated and expanded upon existing evidence, confirming the reliability and stability of CD73 and CD304 as robust MRD markers in childhood BCP-ALL.

## 4. Materials and Methods

### 4.1. Patients and Treatment

This study analyzed a cohort of 98 pediatric patients with leukemia associated immune phenotypes (BCP-ALL) diagnosed and treated at the Pediatric Oncohematology Department of St. George University Hospital in Plovdiv, Bulgaria. FC testing was conducted in the Department of Medical Microbiology and Immunology “Prof. Elissay Yanev” at Medical University-Plovdiv. All patients underwent Berlin–Frankfurt–Münster (BFM)-type induction therapy, with subsequent treatment adjusted based on risk stratification.

### 4.2. Bone Marrow Aspirates

A total of 216 bone marrow samples from 98 children with ALL were analyzed. Leukemic cells were identified in 143 samples (22 diagnostic and 121 MRD-positive samples—a cluster of ≥10 cells with at least two LAIPs), while 73 samples were negative. Additionally, marker expression patterns were evaluated in hematogones from 20 non-leukemic childhood BM aspirates (routinely taken for other conditions). All BM aspirates were processed immediately after receival or up to 48 h of collection to ensure data accuracy.

### 4.3. Panel Design and Sample Procession

Samples were immunophenotyped using a 14-color panel comprising the following fluorochrome-conjugated monoclonal antibodies: CD45-BV480, CD20-APC-H7, CD22-APC, CD86-BV711, CD58-BUV395, CD73-BV421, CD99-BV768, CD44-BV650, CD304-BV605, CD19-PE-Cy7, CD34-PE-CF594, CD38-APC-R700, CD10-PerCP-Cy5.5, and CD123-PE (BD Biosciences, New Jersey, USA, Table S1). Data acquisition was performed on BD FACS Aria III cell sorter (BD Biosciences, CA, USA), and subsequent analysis utilized Infinicyt multi-dimensional software, version 2.0.4 (Cytognos, Salamanca, Spain).

Additionally, all BM aspirates were stained using a routinely used, standardized, Euro flow-based BCP panel containing the following fluorochrome-conjugated antibodies: SYTO41-Pacific Blue, CD45-BV510, CD58-FITC, CD10-PE, CD34-PerCP-Cy5.5, CD38-PE-Cy7, CD19-APC, and CD20-APC-Cy7 (BD Biosciences, CA, USA, Table S2) and sample acquisition was performed on BD FACSLyric clinical cell analyzer (BD Biosciences, CA, USA). The 14-color panel was specifically designed by incorporating additional B-cell maturation and aberrancy markers based on extensive literature reviews that validated their utility in MRD detection, thereby improving the panel’s diagnostic accuracy and clinical relevance.

### 4.4. Gating Strategy

**Figure 10:**
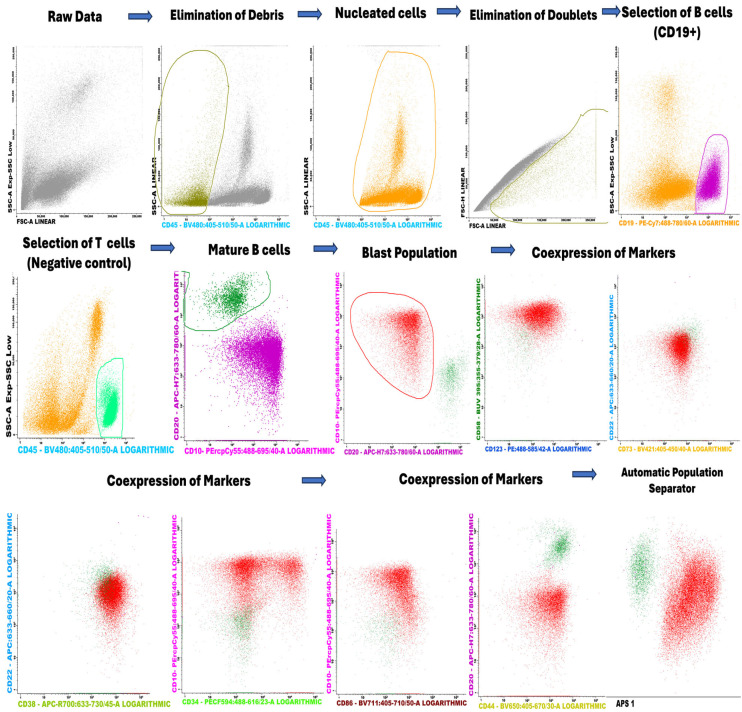
Gating strategy of the lymphocytes in the bone marrow.

A figure demonstrating the gating strategy was added to show how the different lymphocytes were gated from bone marrow (Figure 10). The gating strategy begins with the initial selection of nucleated cells that are Syto41+. From this initial population, B lymphocytes are identified, with T lymphocytes included as a negative control in the selection process. Mature B cells, defined as CD20+/CD10−, are subsequently excluded from the analysis to focus on other lymphocyte populations. The blast cell population is then selected based on the examination of coexpression of specific markers. Finally, the selected populations are visualized using the Automated Cell Separator (APS) tool available in the Infinicyt software, version 10.0.

### 4.5. Multicolor Flow Cytometric Immunophenotyping with 14-Color Panel

Upon receiving the clinical samples, they are processed immediately or up to 48 h after collection to ensure data accuracy and quality. Leukocyte counts are first estimated using an Abacus 380 hematology analyzer (Diatron, Budapest, Hungary) with proprietary CE-certified reagents and control materials. Achieving a detection sensitivity of 0.001% (≤10^−5^) for MRD requires staining and acquisition of a large number of events—over 4 million per sample. Following leukocyte counting using the hemocytometer, approximately 4 × 10^6^ cells are stained in a 50 µL to 100 µL sample volume per tube, with appropriate amounts of the titrated antibodies (the concentration of the antibodies was determined after standard titration studies). For non-leukemic BM samples, the number of events stained was also 4,000,000 and for diagnostic samples was 1,000,000.

Samples undergo a lysis-fixation protocol. They are incubated with the antibodies in the dark at room temperature for 15 min, with antibody concentrations adjusted for larger cell counts. For erythrocyte lysis, BD Lysing Solution (pre-diluted 1:10) is used according to the manufacturer’s instructions (BD Biosciences, CA, USA). The samples are centrifuged at 400× *g* (4 °C, 1400 rpm) for 10 min, supernatants are discarded, and cells are washed with phosphate-buffered saline (PBS). Following a final centrifugation and resuspension in PBS, samples are acquired using the FACS Aria III cell sorter (BD Biosciences, CA, USA). It is equipped with four excitation lasers (405 nm, 488 nm, 561 nm, and 633 nm) and 14 detectors, and facilitates high-throughput acquisition. Event acquisition rates average 3500–4000 cells/second, capturing 3–4 million events for 14-color MRD analysis.

Despite optimized lysing and washing, samples may contain residual debris, including unlysed red cells, reticulocytes, or platelet aggregates, which can inflate cell counts and affect MRD calculations. For this purpose, SYTO41 (Invitrogen Molecular Probes, Oregon, USA), a vital DNA/RNA stain conjugated to Pacific Blue, is included in a supplementary tube containing CD45-BV510, CD19-APC, and CD36-FITC markers (BD Biosciences, CA, USA, Table S3). SYTO41 specifically excludes debris, allowing accurate quantification of approximately 100,000 viable events and precise MRD percentage correction.

The adjusted percentage of MRD is calculated by applying a correction factor. This factor is derived from the ratio of CD19+ B cells in total nucleated cells (Syto41+) from the “SYTO41” tube to CD19+ B cells in all events from the “MRD” tube. The correction factor adjusts for residual debris or non-nucleated cells that could skew MRD measurements. The final corrected MRD percentage is obtained by multiplying the uncorrected MRD percentage by this correction factor.

Files were then exported in FCS format and analyzed by Infinicyt multi-dimensional software, version 2.0.4 (Cytognos, Salamanca, Spain) using a predefined template-based approach.

The study involved evaluating the expression patterns of the new markers in diagnostic and MRD samples. It also examined whether there were statistically significant differences in the mean fluorescence intensity (MFI) between diagnostic blasts and MRD blasts compared to hematogones. Additionally, optimal threshold values were determined to maximize their ability to differentiate between blasts and normal B-cell precursors.

### 4.6. Variable Importance Plot of Biomarkers for Predicting MRD Status Using Random Forest

To investigate the predictive importance of eight biomarkers (CD44, CD304, CD99, CD86, CD73, CD123, CD22, and CD58) in determining MDR status, a Random Forest (RF) classification model [51,52] was applied using the randomForest package in R (version 4.4.3). The model was configured with 100 trees (ntree = 100) and considered a subset of features at each split (mtry = 2) to ensure diversity among trees and mitigate overfitting. The data were split into training (70%) and testing (30%) sets using stratified sampling via the caret package’s createDataPartition function, ensuring proportional representation of MRD classes in both sets. Model performance was assessed using a confusion matrix and key classification metrics, including accuracy, sensitivity, specificity, positive predictive value (PPV), and negative predictive value (NPV). The importance of each biomarker was determined using the Mean Decrease in Accuracy (MDA) and Mean Decrease in Gini (MDG).

## 5. Conclusions

The highest relative frequences of LAIPs were reported for CD99 and CD58, while with lowest relative frequence was CD123. In MRD-positive samples, CD73 showed significantly high differential expression between all stages of hematogones and residual blasts, followed by CD304, CD58, and CD22. CD73 and CD304 were identified as the most reliable among the tested markers for distinguishing both diagnostic and MRD blasts from normal B cell precursors. The threshold values of MFI CD73 in distinguishing diagnostic and residual BCP-ALL blasts from Pre-B-I and Pre-B-II cells are >2930 and >886, respectively. The threshold values of MFI CD304 in discriminating diagnostic and residual BCP-ALL blasts from immature and mature B cells are >14.6 and >112, respectively. In conclusion, further studies will be needed to verify these results and provide more robust statistical power to our observations.

## 6. Patents

One Utility Model owned by the Medical University-Plovdiv and registered in The Patent Office of the Republic of Bulgaria (Registration number 4963/U1/15.11.2024) ‘’System for assessment and analysis of minimal residual disease in childhood precursor acute lymphoblastic leukemia by multiparameter flow cytometry” resulted from the work reported in this manuscript.

## Figures and Tables

**Table 1:** Number of cells, specifically focusing on the mean, median, and standard deviation (SD) for each B-cell group.

Numbers of Cells	Mean	Median	Standard Deviation (SD)
Residual Blasts	2161.64	2288	267.52
Pre-B-I	8537.10	13,128	22,401.90
Pre-B-II	213,539.75	28,774	37,162.67
Immature B Cells	32,416.30	18,517.50	33,343.77
Mature B Cells	56,374.95	43,010	58,503.87

**Table 2:** Model characteristics and classification accuracy of full model. The null model includes only the constant and predicts a baseline probability of 39.8% for MRD = 1. The full model (Enter method) includes all predictors (CD44, CD304, CD99, CD86, CD73, CD123, CD22, CD58).

Model	−2 Log Likelihood	Nagelkerke R^2^	Overall Accuracy (%)	Sensitivity (%)	Specificity (%)
Null Model	150	0	60.2	0	100
Full Model	101.944	0.767	90	92.6	86.3

**Table 3:** Coefficients of the full model. Significant predictors (*p* < 0.05) include CD44, CD304, CD73, CD123, and CD58. The 95% confidence interval (CI) for Exp(B)\text{Exp}(B) Exp(B) was calculated (hypothetical, as not provided in the output, but can be obtained in SPSS 25 via “Options” > “CI for exp(B)”).

Variable	B	S.E.	Wald	*p*-Value	Exp(B)\text{Exp}(B) Exp(B)	95% CI for Exp(B)\text{Exp}(B) Exp(B)
CD58	3.087	0.721	18.333	<0.001	21.911	5.033–95.389
CD304	1.777	0.238	24.372	<0.001	3.245	2.038–5.167
CD73	1.998	0.197	30.932	<0.001	2.997	2.035–4.415
CD123	−0.553	0.233	5.635	0.018	0.575	0.364–0.910
CD44	−0.925	0.360	6.596	0.010	0.397	0.196–0.804
CD22	0.331	0.224	2.192	0.139	1.392	0.897–2.160
CD99	0.220	0.962	0.052	0.819	1.246	0.189–8.227
CD86	−0.199	0.251	0.633	0.426	0.819	0.501–1.340
Constant	−12.512	3.854	10.529	0.001	0	-

**Table 4:** Model characteristics and classification accuracy (Forward Selection). The −2 Log Likelihood decreased progressively, indicating improved model fit with each step. Nagelkerke R^2^ peaked at 0.757 in Step 5, explaining 75.7% of the variance. The Hosmer–Lemeshow test *p*-values indicate poor fit in Step 1 (*p* < 0.001), but good fit in Steps 2–5 (*p* > 0.05), with Step 4 showing the best fit (*p* = 0.942). Step 4 achieved the highest overall accuracy (89.1%), with a balanced sensitivity (91.7%) and specificity (85.0%).

Step	−2 Log Likelihood	Nagelkerke R^2^	Hosmer-Lemeshow Test (*p*-Value)	Overall Accuracy (%)	Sensitivity (%)	Specificity (%)
1	194.315	0.425	<0.001	77.6	87.6	62.5
2	155.196	0.589	0.478	82.1	86	76.3
3	121.942	0.706	0.931	86.1	88.4	82.5
4	112.216	0.736	0.942	89.1	91.7	85
5	105.346	0.757	0.702	88.6	91.7	83.8

**Table 5:** Coefficients of the Best Model (Step 4). Data are based on the “Variables in the Equation” table for Step 4.

Variable	B	S.E.	Wald	*p*-Value	Exp(B)\text{Exp}(B) Exp(B)
CD44	−0.781	0.29	7.243	0.007	0.458
CD304	1.069	0.212	25.386	<0.001	2.911
CD73	0.949	0.166	32.564	<0.001	2.582
CD58	2.907	0.609	22.802	<0.001	18.303
Constant	−11.45	2.414	22.502	<0.001	0

**Table 6:** Results of ROC curve analyses for the reliability of the new markers in differentiating diagnostic blasts from normal B lymphocytes (Pre-B-I, Pre-B-II, immature, and mature B cells in bone marrow).

New Markers	Dg * Blasts Pre-B-I AUC	Dg Blasts Pre-B-II AUC	Dg Blasts Immature B AUC	Dg Blasts Mature B AUC
MFI CD44	0.630	0.770	0.436	0.093
MFI CD304	0.802	0.910	0.905	0.895
MFI CD99	0.409	0.475	0.389	0.284
MFI CD86	0.561	0.548	0.643	0.703
MFI CD73	0.855	0.920	0.850	0.582
MFI CD123	0.543	0.470	0.505	0.652
MFI CD22	0.566	0.791	0.793	0.425
MFI CD58	0.716	0.801	0.789	0.755

* Dg blasts—Diagnostic Blasts.

**Table 7:** Results of ROC curve analyses for the reliability of the new markers in differentiating residual blasts from normal B lymphocytes (Pre-B-I, Pre-B-II, immature, and mature B cells in bone marrow).

New Markers	Residual Blasts Pre-B-I AUC	Residual Blasts Pre-B-II AUC	Residual Blasts Immature B AUC	Residual Blasts Mature B AUC
MFI CD44	0.520	0.601	0.419	0.211
MFI CD304	0.646	0.872	0.914	0.900
MFI CD99	0.539	0.560	0.517	0.371
MFI CD86	0.505	0.502	0.599	0.677
MFI CD73	0.836	0.864	0.836	0.650
MFI CD123	0.417	0.400	0.424	0.531
MFI CD22	0.562	0.849	0.767	0.387
MFI CD58	0.634	0.797	0.799	0.751

## Data Availability

The data presented in this study are available on request from the corresponding author.

## Supplementary Materials

The following supporting information can be downloaded at: https://www.mdpi.com/article/10.3390/ijms26094282/s1.
