# Supplementary material for: Advancing Measurable Residual Disease Detection in Pediatric BCP-ALL: Insights from Novel Immunophenotypic Markers
Source: Int J Mol Sci. 2025 Apr 30;26(9):4282. doi: 10.3390/ijms26094282 (PMC12072110; doi:10.3390/ijms26094282)
Supplement: Supplementary file 1 [file ijms-26-04282-s001.zip › ijms-3407188-supplementary.pdf]

Supplementary Material

Table S1: Major MRD tube with 14-color panel of monoclonal antibodies against leukocyte antigens in BCP-ALL.

**Table S1**				
**Major MRD Tube (14-color)**				
**Marker**	**Fluorochrome**	**Clone**	**Manufacturer**	**Catal. №**
CD58	BUV395	1C3	BD Biosciences	565460
CD73	BV421	AD2	BD Biosciences	562430
CD45	BV480	HI30	BD Biosciences	566115
CD44	BV650	L178	BD Biosciences	743665
CD86	BV711	2331 FUN-1	BD Biosciences	563158
CD99	BV786	TU12	BD Biosciences	743045
CD123	PE	9F5	BD Biosciences	340545
CD34	PE-CF594	581	BD Biosciences	562383
CD10	PERCP-CY5.5	HI10a	BD Biosciences	563508
CD19	PE-CY7	SJ25C1	BD Biosciences	557835
CD22	APC	HIB22	BD Biosciences	562860
CD38	APC-R700	HIT2	BD Biosciences	564979
CD20	APC-H7	2H7	BD Biosciences	560734
CD304	BV605	U21-1283	BD Biosciences	743130

Table S2: 8-color panel of monoclonal antibodies against leukocyte antigens for immunophenotyping in BCP-ALL.

**Table S2**				
**8-color panel**				
**Marker**	**Fluorochrome**	**Clone**	**Manufacturer**	**Catal. №**
CD10	PE	HI10a	BD Biosciences	332776
CD34	PerCP-Cy5.5	8G12	BD Biosciences	347222
CD38	PE-Cy7	HB7	BD Biosciences	335825
CD58	FITC	1C3	BD Biosciences	555920
CD19	APC	SJ25C1	BD Biosciences	345791
CD20	APC-Cy7	L27	BD Biosciences	340908
CD45	BV510	HI30	BD Biosciences	563204

Table S3: Syto tube with monoclonal antibodies against leukocyte antigens for correction of MRD percentage.

**Table S3**				
**Syto41 tube**				
**Marker**	**Fluorochrome**	**Clone**	**Manufacturer**	**Catal. №**
SYTO41	Pacific Blue	-	Invitrogen	S11353
CD19	APC	SJ25C1	BD Biosciences	345791
CD45	BV510	HI30	BD Biosciences	563204
CD36	FITC	CB38	BD Biosciences	561820
